# Microbial diversity and biogeochemical cycling potential in deep-sea sediments associated with seamount, trench, and cold seep ecosystems

**DOI:** 10.3389/fmicb.2022.1029564

**Published:** 2022-10-28

**Authors:** Xiaoyong Zhang, Keyue Wu, Zhuang Han, Zihui Chen, Zhiying Liu, Zuwang Sun, Liyi Shao, Zelong Zhao, Lei Zhou

**Affiliations:** ^1^University Joint Laboratory of Guangdong Province, Hong Kong and Macao Region on Marine Bioresource Conservation and Exploitation, College of Marine Sciences, South China Agricultural University, Guangzhou, China; ^2^Institute of Deep-Sea Science and Engineering, Chinese Academy of Science, Sanya, China; ^3^Liaoning Key Lab of Germplasm Improvement and Fine Seed Breeding of Marine Aquatic Animals, Liaoning Ocean and Fisheries Science Research Institute, Dalian, China

**Keywords:** biogeochemical cycling, deep-sea sediments, high-depth sequencing, metagenomics, microbial communities

## Abstract

Due to their extreme water depths and unique physicochemical conditions, deep-sea ecosystems develop uncommon microbial communities, which play a vital role in biogeochemical cycling. However, the differences in the compositions and functions of the microbial communities among these different geographic structures, such as seamounts (SM), marine trenches (MT), and cold seeps (CS), are still not fully understood. In the present study, sediments were collected from SM, MT, and CS in the Southwest Pacific Ocean, and the compositions and functions of the microbial communities were investigated by using amplicon sequencing combined with in-depth metagenomics. The results revealed that significantly higher richness levels and diversities of the microbial communities were found in SM sediments, followed by CS, and the lowest richness levels and diversities were found in MT sediments. *Acinetobacter* was dominant in the CS sediments and was replaced by *Halomonas* and *Pseudomonas* in the SM and MT sediments. We demonstrated that the microbes in deep-sea sediments were diverse and were functionally different (e.g., carbon, nitrogen, and sulfur cycling) from each other in the seamount, trench, and cold seep ecosystems. These results improved our understanding of the compositions, diversities and functions of microbial communities in the deep-sea environment.

## Introduction

Deep-sea sediment environments contain a diverse array of abundant microorganisms, which play a vital role in biogeochemical cycling (Zhuang et al., 2019). The deep-sea environment contains several different geographic structures with distinct characteristics, including seamounts, trenches, cold seeps, and hot springs (Ingole and Koslow, 2005). Due to the obvious differences in water depths, temperatures, and physico-chemical conditions among the different geographic structures in deep-sea environments, they may have different microbial communities and perform different ecological functions (Levin et al., 2001). Chemosynthetic microorganisms that are enriched in deep-sea hot springs can fix carbon at high rates, which results in higher primary productivity than in other deep-sea regions (McNichol et al., 2018). Another study related to the Yap Trench revealed that the microorganisms in deep-sea sediments played an important role in the weathering process of volcanic materials (Li et al., 2020). Moreover, typical sulfur-oxidizing bacteria have been detected in deep-sea hydrothermal vents with substantial abundances (Ding et al., 2017). Therefore, it is necessary to obtain information on the microbial communities in different deep-sea ecosystems and compare the differences in their capacities for biogeochemical cycling. This information can help us to better understand the underlying mechanisms that regulate microbial ecological processes.

An important issue in microbial ecology investigations is to obtain the compositions of microbial communities in different environmental ecosystems and to link them with ecological processes, such as the cycles of basic chemical elements (Martiny et al., 2006). Determinations of these connections rely on accurate compositional and functional data of microbial communities (Guo et al., 2019). In recent years, the development of high-throughput sequencing technologies, especially amplicon and metagenomics, has provided an effective tool for comprehensively studying the compositions and functions of complex microbial communities in natural ecosystems (Mallick et al., 2017). Amplicon sequencing of barcode genes has been used to determine the composition of complex microbial communities (Lundberg et al., 2013). Meanwhile, the functions of microbial communities can be comprehensively evaluated *via* metagenomic sequencing (Ju and Zhang, 2015). Moreover, combined with in-depth binning analysis, we can obtain microbial genomes without isolation and culture and then accurately determine the relationships among microbial compositions and their ecological functions (Hua et al., 2019). At present, these technical solutions have been applied in a variety of ecosystems, such as rivers (Liu et al., 2020), soils (Su et al., 2017), permafrosts (Hultman et al., 2015), oceans (Delmont et al., 2018), and animal intestines (Xie et al., 2021).

In this study, sediments from seamounts, trenches, and cold seeps in the deep sea of the Southwest Pacific Ocean were collected. The diversity and composition of the microbial communities in these sediments were determined by using high-throughput sequencing based on the 16S rRNA gene. Meanwhile, high-depth metagenomics combined with binning analysis was applied to recover the main microbial genomes in these sediments, which can link the microbial taxa to biogeochemical cycling. Based on the unique water depth and environmental conditions, we expected significant differences in the diversity, composition, and function of the microbial communities in the sediments from different deep-sea ecosystems. In addition, we forecasted the metabolic patterns of the microbial communities from different geographic regions in the deep sea.

## Materials and methods

### Study area and sample collection

**Figure 1:**
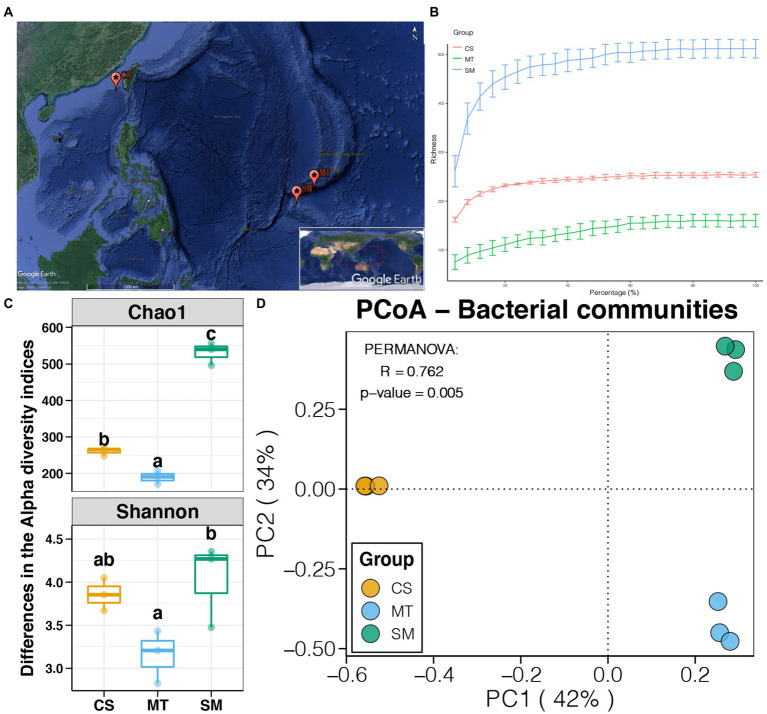
**(A)** Map of sampling sites (Source: Google Earth). **(B)** Rarefaction curves for microbial communities among CS, MT, and SM groups. **(C)** Differences in alpha diversity indices of sediment microbial communities among CS, MT, and SM groups. Different lowercases letters above each box in the same subfigure represent significant differences between groups (Tukey’s HSD test, *p* < 0.05). **(D)** Principal coordinate analysis (PCoA) and PERMANOVA test of the sediment microbial communities among CS, MT, and SM groups.

Sediment samples from marine trenches (11.65°N, 142.23°E, water depth of 6,010 m, MT); cold seeps (22.16°N, 119.29°E, water depth of 1,120 m, CS), and seamounts (flank, 10.02°N, 140.20°E, water depth of 1,555 m, SM) were collected from the Southwest Pacific Ocean during research that was conducted by the Chinese Academy of Science during several cruises between July 2016 and June 2019 (Figure 1A). The sediments were collected using a box corer attached to a Hadal Lander. The sediment samples were immediately recovered onboard, and three subsamples (named MT1, MT2, and MT3, CS1, CS2, and CS3, SM1, SM2, and SM3, respectively) of the three sediments (e.g., MT, CS, and SM) were then placed into sterile plastic corers and stored at −80°C for DNA extractions.

### DNA extraction and sequencing

The sediment samples were thawed on ice, and only the interior sections were selected for DNA extraction to avoid potential contamination. Approximately 500 mg of sediment from each sample was used, and the DNA was extracted using the FastDNA SPIN Kit for Soil (MP Biomedicals, Santa Ana, CA, United States) according to the manufacturer’s instructions. The integrity of the genomic DNA was verified by agarose gel electrophoresis, while its concentration and purity were measured with a Nanodrop 2000 spectrophotometer (Thermo Fisher Scientific, Waltham, MA, United States) and Qubit 3.0 fluorometer (Thermo Fisher Scientific). The genomic DNA was stored at −20°C for subsequent sequencing.

The compositions of the sediment microbial communities were assessed by using 16S rRNA amplicon sequencing. The V3–V4 hypervariable regions of the 16S rRNA gene were amplified using the primers 341F (5′-CCTACGGGNGGCWGCAG-3′) and 806R (5′-GACTACHVGGGTATCTAATCC-3′; Klindworth et al., 2013). The amplification and library construction procedures were performed according to the manufacturer’s instructions. The constructed libraries were sequenced on an Illumina MiSeq PE 300 × 2 platform (Illumina Inc., San Diego, CA, United States) at Majorbio, Shanghai, China.

Before the metagenomic sequencing, three DNA samples that were extracted from the sediments in the same deep-sea geographic region were equally mixed. Then, 0.1 μg of DNA per geographic region was used as the input material for sequencing preparations. Multiple displacement amplification (MDA; Yilmaz et al., 2010) was used for DNA amplification. Briefly, the extracted DNA was added to phageome DNA as spike-in DNA (approximately 5% of the phageome DNA amount). MDA was performed for 3.0–10.0 ng of phageome DNA at 45°C for 180 min using an EquiPhi29 (Thermo Fisher Scientific; Stepanauskas et al., 2017) and an Exo-resistant random primer (Thermo Fisher Scientific; Kiguchi et al., 2021). The sequencing libraries were generated using the NEBNext® Ultra™ DNA Library Prep Kit for Illumina (NEB, United States) by following the manufacturer’s recommendations. The metagenomic sequencing was performed on the Illumina NovaSeq 6000 platform (Illumina Inc., San Diego, CA, United States) at Majorbio, Shanghai, China. After sequencing, the raw data with low quality (e.g., Phred scores lower than 30, containing ambiguous bases or sequence lengths shorter than 150 bp) were removed by using the NGS QC Toolkit to generate clean data before the bioinformatics analysis (Patel and Jain, 2012). Approximately, 120 Gb (giga base pairs) of metagenomic data were generated for each sediment type that was obtained from the deep-sea geographic region.

### Taxonomy and functional annotation

For the amplicon data, VSEARCH 2.18 was used to perform paired-end merging, quality filtering, and dereplication. Then, the nonredundant sequences were inferred to be exact amplicon sequencing variants (ASVs) by using the unoise3 plug-in unit in the USEARCH 10.0 software (Edgar, 2010). The taxonomies were assigned (e.g., kingdom, phylum, class, order, family, genus, and species) by the USEARCH SINTAX algorithm in the RDP training database v18. The ASVs that mapped to mitochondria or chloroplasts were discarded. An ASV abundance table was constructed and normalized by using a standard number of tags based on the sample with the least number of tags (38,316).

For the metagenomic data, the clean data from each library were assembled into contigs using MEGAHIT with the default parameters (Li et al., 2015). The potential functional genes were predicted from the contigs by using prodigal v2.6.3 with the “-p meta” option (Hyatt et al., 2010). CD-hit was then used to remove the redundant sequences (90% coverage and 95% identity) among the samples (Fu et al., 2012). The gene functional annotations (e.g., COG and CAZyme) were made by using an eggNOG-mapper BLASTP search in eggNOG 5.0 (Huerta-Cepas et al., 2019) with an e-value ≤1 × 10–3 and score ≥ 60. In each sample, the clean reads were used to map back to the predicted genes by using Salmon with the default parameters (Patro et al., 2017) to obtain the abundance of each gene (Bushnell, 2014). The biogeochemical pathways were inferred from the metagenomic data and compared using DiTing (Xue et al., 2021).

### Metagenomic binning

To obtain the genomes of the potential participants in biogeochemical cycling, the contigs in each metagenomic library were utilized to conduct binning using tetranucleotide frequency and coverage values using MetaWRAP v1.3 (Uritskiy et al., 2018). The completeness and contamination of the dereplicated bins were estimated using CheckM v1.0.7 (Parks et al., 2015). The bins with completeness higher than 50% and contamination lower than 10% were retained and dereplicated using dRep v2.6.2 (Olm et al., 2017) with the following parameters: -sa 0.95 -nc 0.30 -comp 50 -con 10. The taxonomic annotations were completed using GTDB-Tk based on the Genome Taxonomy Database (GTDB; Chaumeil et al., 2020). A maximum-likelihood phylogenetic tree was established based on the 120 bacterial marker genes identified in GTDB-Tk. The abundance of bins was calculated with the Quant_bins module (default parameters) in MetaWRAP, which mapped the clean reads to the contigs and calculated the length-weighted average of the contig coverage for each bin in each sample. Gene predictions for all remaining bins were conducted using Prodigal v 2.6.3 with the default parameters (Hyatt et al., 2010). To annotate the genes related to biogeochemical cycling, these recognized genes were aligned against the Kyoto Encyclopedia of Genes and Genomes (KEGG) database (*e*-value ≤ 0.001) using KofamScan (Aramaki et al., 2020).

### Statistical analysis

The alpha diversity indices of the sediment microbial communities, including the Chao1 and Shannon indices, were calculated by using the vegan R package. Boxplots based on the alpha diversity indices were created, and the differences among different ecosystems were analyzed using Tukey’s honest significant difference (HSD) test. The variations in the microbial community compositions of the sediments among different ecosystems were evaluated by using principal coordinate analysis (PCoA) and PERMANOVA using the “vegan” package in R v4.0.2. The linear discriminant analysis effect size (LEfSe) was used to identify the significantly different abundances of microorganisms among the sediments from different ecosystems (Segata et al., 2011). Bar graphs were used to compare the abundance differences of the functional terms of the microbial communities among the sediments from SM, MT, and CS. The abundances of each bin were compared by using bar graphs. Finally, a dot chart was drawn to indicate the presence of specific genes related to biogeochemical cycling in different bins. All charts in this study were created by the “ggplot2” package in the R v4.0.2 platform.

## Results

### Variations in the diversity of microbial communities

In this study, a total of nine sediment samples, three subsamples belonging to each of three different deep-sea ecosystems (e.g., SM, MT, and CS), were collected from the Southwest Pacific Ocean. High-throughput sequencing based on the 16S rRNA gene was performed to obtain their microbial communities. A total of 402,556 high-quality sequences, which ranged from 38,316 to 54,010 in each sample, were obtained. These sequences were clustered into 900 OTUs, and the alpha diversities of the microbial communities were calculated. Rarefaction curves based on the richness for all three deep-sea ecosystems were close to the horizontal state (Figure 1B), demonstrating that the amount of sequencing data for each sample was sufficient to reflect the intact microbial communities. As shown in Figure 1C, significant differences in the alpha diversity indices were found among sediments from the different ecosystems (Tukey’s HSD test, *p* < 0.05). The highest and lowest Chao1 and Shannon indices were observed in the SM and MT sediments, respectively. These findings indicated that the richness and diversity of the sediment microbial communities were higher in seamounts but were lower in trenches.

**Figure 2:**
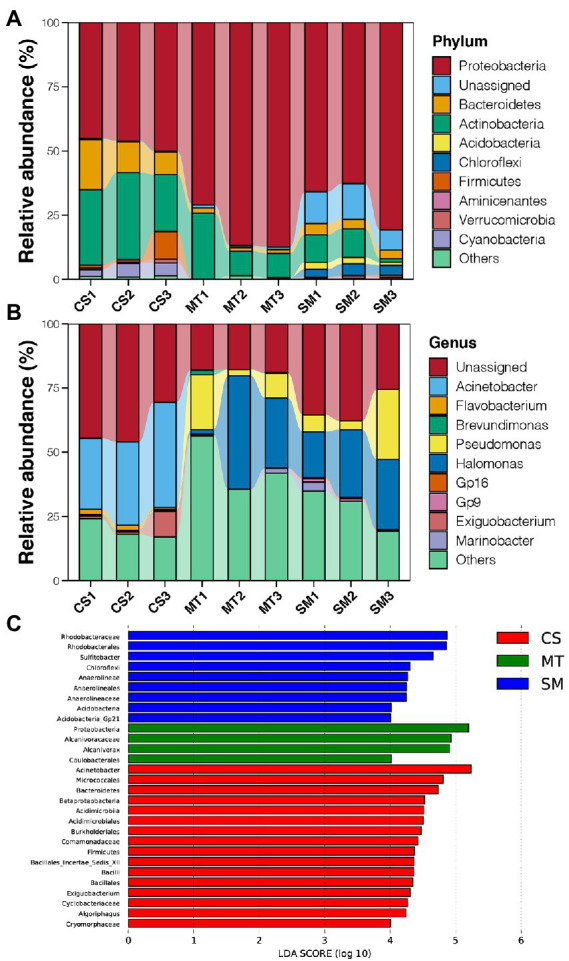
**(A)** Relative abundances of dominant bacterial phyla in sediments of CS, MT, and MS groups. **(B)** Relative abundances of dominant bacterial genera in sediments of CS, MT, and MS groups. **(C)** The LEfSe analysis results of sediment microbial communities among CS, MT, and MS groups. Linear discriminant analysis (LDA) score was used to discover biomarkers in different ecosystem groups. The threshold value was log10 (LDA score) > 4.

Principal coordinate analysis was used to evaluate the differences in the sediment microbial communities among different deep-sea geographic regions, and the results are shown in Figure 1D. The sediment microbial communities from different geographic regions were clustered separately in the PCoA graph. The first two PCs explained 76% of the total variations in the sediment microbial communities, which were 42 and 34% for PC1 and PC2, respectively. Moreover, the PERMANOVA results revealed that the sediment microbial communities were significantly different among the CS, MT, and SM groups (*p* < 0.05). According to the taxonomic annotations, Proteobacteria was the dominant bacterial phylum in the deep-sea sediments, which occupied 70–80% of the microbial communities in the MT and SM samples but only 50% in the CS samples (Figure 2A). Actinobacteria and Bacteroidetes were the second and third most abundant bacterial phyla in the deep-sea sediments, respectively, which had higher abundances in the CS samples than in the MT and SM samples (Figure 2A). In addition, Aminicenantes and Firmicutes were observed in the CS sediments, while relatively higher abundances of Acidobacteria and Chloroflexi were observed in the SM samples (Figure 2A). Moreover, a considerable proportion (11.38%) of the 16S sequences in the SM sediments were unassigned, which indicated that the seamount sediments might contain unknown bacterial phyla.

At the genus level, Acinetobacter was dominant in the CS sediments, while *Halomonas* and *Pseudomonas* dominated in the MT and SM sediments (Figure 2B). In addition, there was a considerable proportion of unassigned sequences at the genus level, which suggested that the current database still had a large gap in its coverage of bacteria from deep-sea environments (Figure 2B). LEfSe analysis was conducted to recognize the potential biomarkers for microbial communities among different deep-sea ecosystems (Figure 2C). The genus *Acinetobacter* belonging to the Proteobacteria phylum (33.63% vs. 0.01 and 0.02%), the order Acidimicrobiales belonging to the Acidimicrobater phylum (6.49% vs. 0.00 and 0.04%), the genus *Exiguobacterium* belonging to the Firmicutes phylum (3.89% vs. 0.00 and 0.01%), and the genus *Algoriphaus* (3.79% vs. 0.60 and 0.00%) and family Cryomorphaceae belonging to the Bacteroidetes phylum (2.21% vs. 0.00 and 0.01%) were found to be more abundant in the CS samples when compared to the MT and SM samples. For the SM sediments, the genus *Sulfitobacter* belonging to the Proteobacteria phylum (8.71% vs. 4.36 and 0.00%), the family Anaerolineae belonging to the Chloroflexi phylum (3.51% vs. 0.00 and 0.02%), and the genus Gp21 belonging to the Acidobacteria phylum (0.26% vs. 0.01 and 0.00%) were more abundant. In contrast, only the genus *Alcanivorax* (15.37% vs. 0.00 and 0.00%) and order Caulobacterales belonging to Proteobacteria (2.09% vs. 0.65 and 0.01%) were more abundant in the MT sediments.

### Function profiles of microbial communities

**Figure 3:**
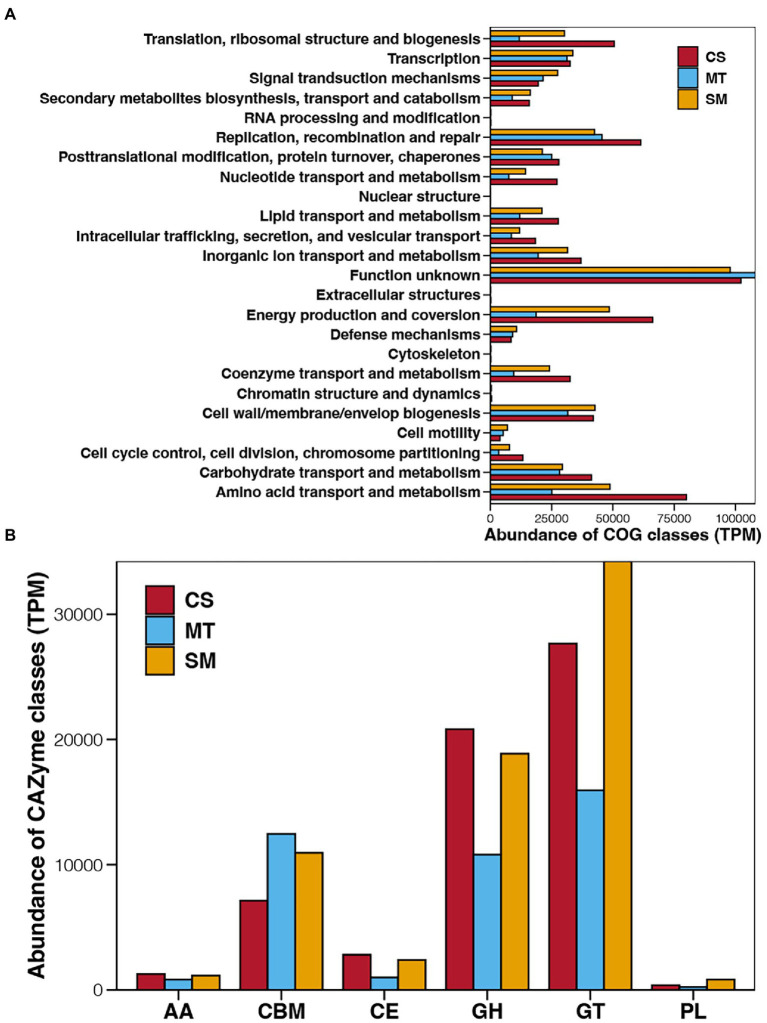
Abundances of COG **(A)** and CAZyme **(B)** classes in sediments from CS, MT, and MS groups. AA, auxiliary activity; CBM, carbohydrate-binding module; CE, carbohydrate esterase; GH, glycoside hydrolases; GT, glycosyl transferase; and PL, polysaccharide lyase.

To establish the functions of the microbial communities in deep-sea sediments, the identified genes were first assigned to COG, which clustered the functional genes into 24 COG classes with different functions (Figure 3A). Regardless of whether the genes were derived from CS, MT, or SM sediments, the COG class with the highest abundance was “Function unknown,” which suggested limited knowledge of deep-sea microbial functions. The other main COG classes for the deep-sea sediments were “Energy production and conversion” and “amino acid transport and metabolism,” which had higher abundances in the CS and SM groups compared to other COG classes but relatively lower abundances in the MT sediments. Except for the “Function unknown” term, the abundances of the COG classes in the MT sediments were generally lower than those in the CS or SM sediments, among which “Replication recombination and repair” was dominant in the MT sediments.

The function genes related to carbohydrate-active enzymes were annotated by CAZyme database and their abundance of different CAZyme classes among different deep-sea sediments are shown in Figure 3B. Glycosyl transferase was the dominant CAZyme class in the deep-sea sediments, followed by glycoside hydrolases and carbohydrate-binging modules. In contrast, the abundances of carbohydrate esterases, auxiliary activities, and polysaccharide lyases in deep sea sediments were relatively low. Among different ecosystems, the abundances of auxiliary activities, carbohydrate esterases, and glycoside hydrolases were highest in CS sediments, second in SM, and lowest in MT group, while the abundance order of carbohydrate-binging modules were just the opposite. Moreover, the abundances of glycosyl transferase and polysaccharide lyases were highest in SM sediments, second in CS, and lowest in MT group.

### Biogeochemical cycling potential

**Figure 4:**
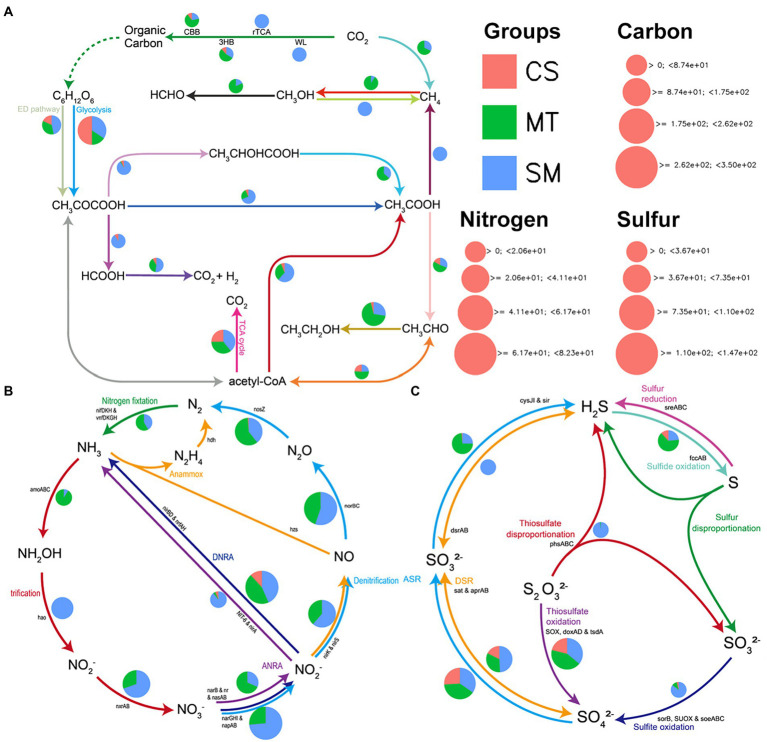
The patterns of genes related to carbon **(A)**, nitrogen **(B)**, and sulfur **(C)** cycling in sediments from CS, MT, and SM groups.

The biogeochemical functions and cycling of carbon, nitrogen, and sulfur in the sediment microbial communities among the different ecosystems were evaluated and compared. The abundances of genes related to carbon in the CS, MT, and MS groups are compared in Figure 4A. The most abundant genes detected in the CS sample were from genes associated with glycolysis, while the other carbon cycle capacities were relatively weak. Genes related to methanogenesis were detected only in the SM sediments, and the MT samples had a strong ability to fix methane. For carbon dioxide fixation, the microbial communities in MS mainly functioned *via* the rTCA and WL pathways, while the CBB and 3HB pathways were more abundant in the MT and CS sediments. In addition, the carbon in the SM sediments was mainly used to produce methane through the acetic acid production pathway, while the synthesis of ethanol by acetyl-CoA was dominant in the MT sediments. For the genes related to nitrogen cycling, the abundances of genes related to nitrification/denitrification and nitrogen fixation were higher in the SM and MT sediments, respectively (Figure 4B). However, almost no nitrogen cycle-related genes were detected in the CS sediments (Figure 4B). Similar to the nitrogen cycle gene, the abundances of sulfur cycle-related genes were also higher in the SM and MT sediments than in the CS sediments (Figure 4C). In the SM and MT sediments, the reduction of sulfates to produce sulfides was mainly mediated by the DSR and ASR pathways, respectively (Figure 4C). Moreover, the sulfite oxidation and thiosulfate disproportionation abilities were more powerful in the SM sediments (Figure 4C).

**Figure 5:**
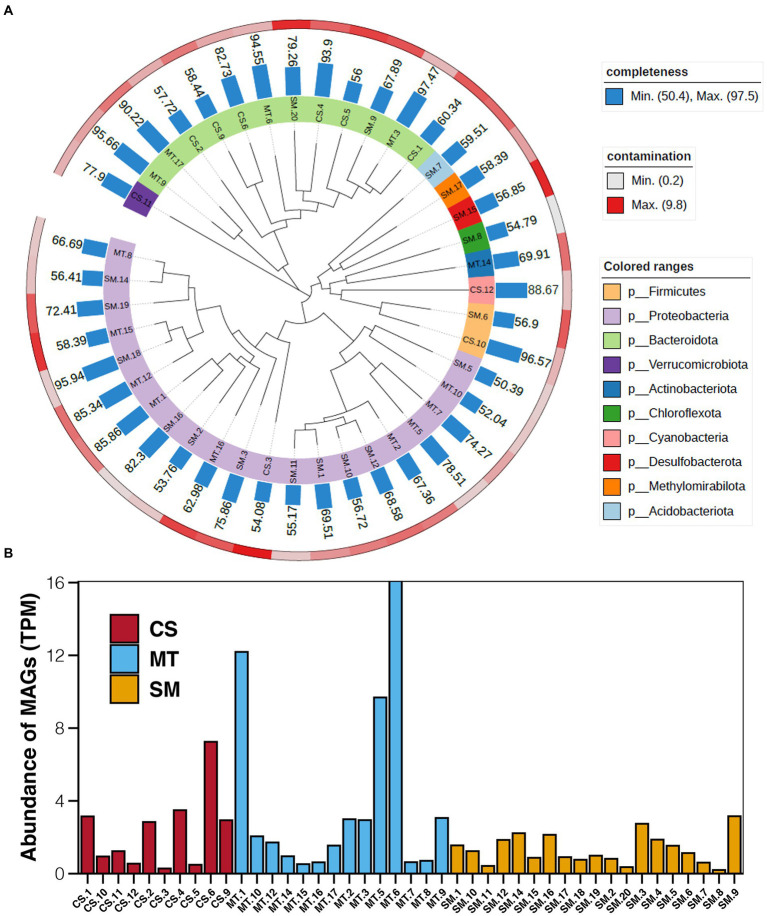
**(A)** Maximum-likelihood phylogenetic tree showing the affiliation, completeness, and contamination of retrieved MAGs. **(B)** Abundance of MAGs in sediments from CS, MT, and SM. The unit of MAG abundance is TPM, the average read coverage of a bin per million mapped reads.

Based on the results of binning, a total of 43 bins with more than 50% completeness and less than 10% contamination were obtained, which were 10, 14, and 19 in CS, MT, and SM sediments, respectively. Among these bins, Proteobacteria and Bacteroidota were the most numerous bacterial phyla. Bins belonged to the Proteobacteria phylum were mainly present in MT and SM sediments, while bins uncovered in CS sediments were generally Bacteroidota (Figure 5A). The abundances of these bins were calculated, and the results are shown in Figure 5B. In CS sediments, bin of CS.6 (SXYR01) had the highest abundance, followed by CS.1 (Flavobacterium), CS.2 (UBA955), CS.4 (AKYH767), and CS.9 (Sediminibacterium). In MT sediments, the most abundant bin was MT.6 (Algoriphagus), followed by MT.1 (HB2-32-21) and MT.5 (Hyphomonas). In SM sediments, no bins showed absolute dominance in terms of abundance, although the greatest number of bins was uncovered.

**Figure 6:**
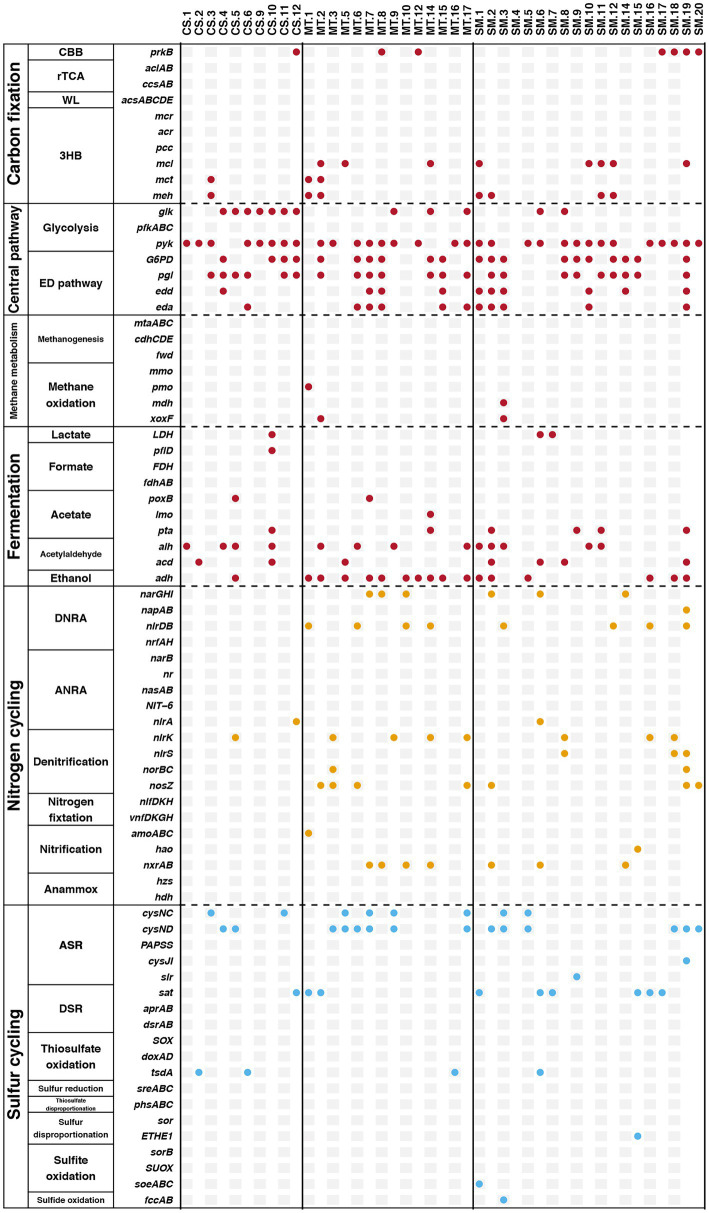
The presence of specific genes related to biogeochemical cycling of carbon (red), nitrogen (yellow), and sulfur (blue) in retrieved bins.

Furthermore, genes related to biogeochemical cycling were identified in these retrieved bins, and the results are shown in Figure 6. Genes for the glycolysis and ED pathways were identified in most bins, which indicated that these central carbon cycling pathways were accomplished by diverse bacteria. The methane oxidation pathway was found in bins MT.1, MT.2, and SM.3, which all belonged to the Proteobacteria phylum. The number of bins carrying the adh gene for ethanol synthesis in the MT (10) sediments was much higher than those in the CS (1) and SM (6) sediments, which was consistent with the powerful ethanol synthesis revealed by the contigs results (Figure 4A).

## Discussion

The microorganisms present in deep-sea sediments remain largely unknown due to the complexity of the sediment communities, and the special environmental conditions restrict the isolation of deep-sea microorganisms (Sogin et al., 2006). The distinct geochemical environments in different geographic structures of the deep sea may select unique microbial communities (Zhang et al., 2021). These microorganisms could play different roles in utilizing inorganic nutrients and transforming the organic compounds that are involved in deep-sea biogeochemical cycles (Huang et al., 2019). As a culture-independent technology, the metagenomics of marine sediments has revealed a broad diversity of uncultured microorganisms and provided insights into their metabolic capabilities (Wang et al., 2016; Zhang et al., 2016). Here, we presented a glimpse of the differences in the sediment microbial communities among different geographic structures in deep-sea environments. Moreover, the assembly and binning of the in-depth metagenomes recovered many uncultured genomes that enlarged the tree of life and improved the understanding of biogeochemical cycling in deep-sea sediments.

The benthic community compositions in marine environments are depth stratified and reflect environmental gradients that are correlated with depth, such as temperature, oxygen content, light level, and pressure (Clark et al., 2010). In this study, we observed significant differences in the diversities and compositions of the microbial communities within the CS, MT, and SM sediments (Figure 1). Significantly higher richness and diversity of the microbial communities were observed in the SM and CS sediments compared to those in the MT samples, which was consistent with the lower depths of SM (~ 1,500 m) and CS (~ 1,100 m) than MT (~ 6,000 m). With increasing water depth, the seawater pressure gradually increases, and the microbial diversity decreases due to increased selection in more extreme environments (Wakeham et al., 2007). A negative correlation between biodiversity and water depth has been observed in multiple previous studies (Varliero et al., 2019; Wu et al., 2020; Zhang et al., 2021). Similar to the alpha diversity indices, the beta diversity, as shown in the PCoA plot in Figure 1D, also demonstrated significant variations in the bacterial communities among SM, CS, and MT. Remarkable shifts in the bacterial communities with the water depth gradient were also found in studies of multiple marine areas regardless of whether they were associated with water or sediment columns (Yang et al., 2019, 2021). The results of present and previous studies both confirmed the vital effects of water depth on the diversity and composition of bacterial communities in marine environments.

A comparison of the results between the present and previous studies on the abundances of the main bacterial phyla was further performed. In agreement with our results, the presence of Proteobacteria, as the dominant bacterial phylum in deep-sea environments, has been proven by multiple studies (Cui et al., 2019; Peoples et al., 2019; Serrano et al., 2021). Comparable relative abundances of Actinobacteria and Bacteroidetes were also detected in other CS systems in the South China Sea (Cui et al., 2019). However, both Firmicutes and Chloroflexi represented approximately 15% in other CS systems in the South China Sea but nearly disappeared in this study (Cui et al., 2019). For the MT sediments, Proteobacteria and Actinobacteria were the top two bacterial phyla, which occupied over 95% of the microbial communities (Figure 2A). In the sediments obtained from a seamount in the Mariana volcanic arc, Firmicutes was the dominant bacterial phylum, which was followed by Proteobacteria (Liu et al., 2017). However, Firmicutes was nearly absent in the MS sediments obtained in the present study and was replaced by Actinobacteria, Bacteroidetes, Acidobacteria, and Chloroflexi (Figure 2A). In contrast, although Proteobacteria was also the most dominant phylum in the sediments from other trenches, the other microorganisms with high relative abundances were quite different from our results (Peoples et al., 2019). In this work, Actinobacteria was the second most abundant bacterial phylum in the MT sediments (Figure 2A), while Thaumarchaeota, Bacteroidetes, Planctomycetes, and Chloroflexi were found to have substantial abundances in the sediments from hadal trenches (Peoples et al., 2019). These results indicated that the compositions of the microbial communities in deep-sea sediments varied greatly among and within different ecosystems, which were probably affected by the habitat heterogeneity among different geographic locations and niche differentiation at the local scale.

It was noteworthy that Acinetobacter was the dominant genus in the CS sediments (Figure 2B). *Acinetobacter* is a gram-negative coccobacillus that has been recognized as an organism of questionable pathogenicity to an infectious agent of importance to hospitals worldwide for decades (Munoz-Price and Weinstein, 2008). In addition to being implicated in a wide spectrum of infectious diseases, *Acinetobacter* is more concerning because it has multiple resistance to most available antimicrobial agents (Manchanda et al., 2010). In 2017, the World Health Organization recognized carbapenem-resistant *Acinetobacter baumannii* as the critical, number 1 priority, clinically significant pathogen (World Health Organisation, 2017). The high abundance of *Acinetobacter* that was revealed in the CS sediments from our study suggested that CS could be one of the reservoirs and sources of *Acinetobacter*. Unfortunately, no MAG belonged to *Acinetobacter* was obtained in our study by the binning technology (Figure 5A), which prevented us from deeper comparisons. The relationship between Acinetobacter in the clinical and CS environments requires further detailed investigations and comparisons.

Aa a central component of the ocean’s biological carbon pump, organisms in surface water can fix carbon to form particulate organic matters, and then sink to seafloor for transporting carbon and energy to abyssal depths (Poff et al., 2021). In deep-sea sediments, these sinking particles are further decomposed and transformed, which in turn stores carbon in the sediment (Koeve, 2005). For CAZYyme classes, glycoside hydrolases and carbohydrate esterases were responsible for degrading carbohydrates (Drula et al., 2022), and they were more abundant in CS sediments compared to SM and MT (Figure 3B). This result suggested CS ecosystem could possess stronger ability of carbon sequestration than MT and SM sediments. Except for carbon cycle, nitrogen cycle is of great significance to maintain the balance of marine ecosystems (Hutchins and Capone, 2022). Nitrification and denitrification are a pair of ecological processes that regulate the balance of ammonium and nitrate mainly mediated by aerobic microorganisms (Barnard et al., 2005). Although previous studies reported the influences of temperature, pH, and nutrients on nitrification and denitrification, oxygen is the most important factor for these processes (Ji et al., 2018). More powerful nitrification and denitrification have been observed in areas closer to the sea surface with higher dissolved oxygen (Sun and Ward, 2021). Stronger nitrification and denitrification capacities in SM found in this study (Figure 4B) are probably due to the lower water depth of SM compared with MT and CS. In addition, higher abundance of genes related to nitrogen fixation in MT compared with SM and CS (Figure 4B) indicated that high level of N_2_ could release in the MT ecosystem. Arising nitrogen from the decomposition of organic matter in deep-sea trenches, such as the Cariaco Trench (Richards and Benson, 1961) and the Mariana Trench (Liu and Peng, 2019), which could support the powerful nitrogen fixation in MT ecosystem. Moreover, sulfur compounds are used as both electron donor and acceptor by deep-sea microorganisms for energy conservation (Yamamoto and Takai, 2011). Most of previous studies about sulfur cycle in deep-sea ecosystems focused on the sulfur oxidation at hydrothermal vents (Sievert et al., 2007, 2008; Zeng et al., 2021). Our findings in this study provided evidence of the sulfur reduction in other deep-sea ecosystems (Figure 4C).

In this study, in-depth metagenomics combined with binning technology was used to explore the relationships among key microorganisms and the carbon, nitrogen, and sulfur metabolic pathways in different deep-sea geographic structures. Compared with traditional PCR-based approaches, metagenomic analyses result in greater sequence depths and also provide an overview of genetic capabilities (Zeyaullah et al., 2009). The results of the present study showed an important phenomenon in which certain biochemical processes in deep-sea sediments could be conducted by various microbial groups. Genes involved in the rTCA, WL, and methanogenesis pathways in carbon cycling were detected in the results based on contigs but were not found in any bins. It should be noted that these genes were detected only in the contigs from the SM sediments (Figure 4A). For the genes involved in nitrogen and sulfur cycling, many genes were detected in the contigs that were not observed in the bins (Figure 6). Moreover, the cases of SM.9 (Flavobacteriaceae), MT.6 (Algoriphagus), and CS.6 (Chitinophagaceae) deserve to be mentioned, which had the highest abundances in the SM, MT, and CS sediments, respectively (Figure 5B). All of them belong to the Bacteroidota phylum, which has been frequently detected in diverse environments, including soil (Delgado-Baquerizo et al., 2018), sediments (Probandt et al., 2017), sea water (Royo-Llonch et al., 2017), and the guts and skins of animals (Cuskin et al., 2015; Cuscó et al., 2017). In these diverse ecological niches, Bacteroidota are increasingly regarded as specialists for the degradation of high molecular weight organic matter, e.g., proteins and carbohydrates (Li et al., 2017). The recent sequencing of Bacteroidota genomes also confirmed the presence of numerous carbohydrate-active enzymes covering a large spectrum of substrates from plant, algal and animal origins (Han et al., 2009; Del Rio et al., 2010; Qin et al., 2010). These results obtained by previous studies can be mutually confirmed with the multiple carbon cycling-related genes that were detected in SM.9, MT.6, and CS.6, indicating the potential carbon metabolism by Bacteroidota in deep-sea sediments.

Here, we further assigned six Bacteroidota (SM.20, MT.3, MT.9, MT.17, CS.4, and CS.5) as potentially metabolizing the nitrogen and sulfur cycles and coupling the electron flow from organic matter to the reduction of nitrate and sulfate. Bacteroidota genomes appear to be highly plastic and are frequently reorganized through genetic rearrangements, gene duplications, and horizontal gene transfers, which are features that could have driven their adaptation to distinct ecological niches (Francois et al., 2011). Horizontal gene transfer, a process mediated by mobile gene elements, such as plasmids, integrons, and transposons, has been widely investigated due to its role in the acquisition and spread of antibiotic resistance genes (Gillings, 2013; Mikalsen et al., 2015). Evidence is accumulating that certain environmental characteristics, such as the organic matter content, shape the compositions of the microbial communities in diverse environments (Patel et al., 2014). Horizontal gene transfer can provide microbes with tools to degrade otherwise refractory substances (Hehemann et al., 2012). Thus, the metabolic potential of Bacteroidota may be due to horizontal gene transfer and selection pressure from the extreme deep-sea environment. This partially explained why the distribution of Bacteroidota is more abundant in the organic matter-rich ecosystems (e.g., SM and CS) compared to the oligotrophic environment (e.g., MT; Figure 2A). However, it should be noted that the metabolic pathways analyzed in this study were based on genomic DNA analysis, such that our interpretation implies the functional capabilities of these microbiomes rather than their actual activities. Further studies directly based on RNA, protein or metabolite levels are necessary to further explore the active functions in different mariculture systems.

In summary, sediments from SM, MT, and CS located in the South China Sea were collected and analyzed by high-throughput sequencing technologies. Based on the amplicon sequencing of 16S rRNA gene, significant differences in the diversity and composition of microbial communities were observed among the SM, MT, and CS sediments. In addition, the metagenomics approach unveiled the power of biogeochemical cycling of microbial communities in different deep-sea sediments. The results appeared that genes involved in most of the biogeochemical cycle pathways were rarely or almost absent in the CS sediments. In contrast, SM sediments possessed the most diverse biogeochemical cycle genes, and some pathways were only detected in MT sediments. Moreover, resources of functional bacteria participated in the biogeochemical cycling were recognized by binning analysis. Among them, Proteobacteria and Bacteroidota were most dominant, meanwhile, some of them were novel bacteria without accurate taxonomic annotation at species or genus levels. Overall, such information can not only improve the understanding of the deep-sea microbial communities, but also help to explain the molecular mechanism of biogeochemical cycling in the deep sea using a combination of bioinformatic technology.

## Data availability statement

The datasets presented in this study can be found in online repositories. The names of the repository/repositories and accession number(s) can be found at: https://www.ncbi.nlm.nih.gov/, PRJNA820529; https://www.ncbi.nlm.nih.gov/, PRJNA820494.

## Author contributions

LZ and ZZ designed the research. XZ and LZ obtained the funding. ZH, ZC, LS, and ZL analyzed the data. XZ, KW, and ZS wrote the manuscript. All authors contributed to the article and approved the submitted version.

## Funding

This study was funded by the National Key R&D Program of China (2018YFD0900802) and the China-ASEAN Maritime Cooperation Fund (CAMC-2018F).

## Conflict of interest

The authors declare that the research was conducted in the absence of any commercial or financial relationships that could be construed as a potential conflict of interest.

## Publisher’s note

All claims expressed in this article are solely those of the authors and do not necessarily represent those of their affiliated organizations, or those of the publisher, the editors and the reviewers. Any product that may be evaluated in this article, or claim that may be made by its manufacturer, is not guaranteed or endorsed by the publisher.
